# Clinical Research on the Relation Between Body Mass Index, Motilin and Slow Transit Constipation

**DOI:** 10.4021/gr2010.02.168w

**Published:** 2010-01-20

**Authors:** Hong Bin Chen, Yue Huang, Hui Wen Song, Xiao Lin Li, Song He, Jia Tia Xie, Chun Huang, Sheng Jun Zhang, Jia Liu, Ying Zou

**Affiliations:** a Department of Gastroenterology, Sanming First Affiliated Hospital of Fujian Medical University, Sanming 365000, China; b Department of Gastroenterology, the 2nd Affiliated Hospital of Chongqing Medical University, Chongqing 400010, China; c Department of Nuclear Medicine, Sanming First Affiliated Hospital of Fujian Medical University, Sanming 365000, China; d Department of Radiology, Sanming First Affiliated Hospital of Fujian Medical University, Sanming 365000, China

**Keywords:** Body Mass Index, Motilin, Slow Transit Constipation

## Abstract

**Background:**

Constipation is a common clinical symptom but its etiology remains unknown. The aims of the study are to discuss the relation between body mass index (BMI), motilin and the slow transit constipation (STC).

**Methods:**

A total of 178 patients with STC and 123 healthy volunteers as controls were divided into three groups according to the BMI, group A (BMI <20), group B (BMI 20-25), and group C (BMI > 25). Fasting and one hour postprandial plasma motilin were measured and the results were analyzed.

**Results:**

There was significant difference in the constituent ratio between STC patients and healthy controls (p < 0.05). The percentage of group A, B and C in STC patients was 49.4% (88/178), 23.0% (41/178) and 27.6% (49/178), respectively; and group A had a higher percentage. Plasma motilin of fasting and one hour postprandial in STC patients of group A was significantly lower than that of group B and C (p < 0.05), but there was no difference between group B and C (p > 0.05). There was no significant difference in the results of plasma motilin of fasting and one hour postprandial among the three groups of healthy controls (p > 0.05). Plasma motilin of fasting and one hour postprandial in STC patients of group A was significantly lower than those healthy controls of group A (p < 0.05). The same results of plasma motilin of fasting and one hour postprandial could be seen in group B and C, respectively (p < 0.05).

**Conclusions:**

A higher proportion of low BMI sufferers was found in the STC patients. The reason may be related to the lower release of the plasma motilin.

## Introduction

Constipation is a common condition, affecting up to 27% of Americans [1] and resulting in more than 2 million physician visits annually [2]. It is most prevalent in women and people over the age of 65 years [1, 3]. Studies have shown that constipation has a negative effect on the individual’s quality of life [2, 4]. The etiology for constipation is often multifactorial, possibly the sign of an underlying organic disease [5, 6] and increases the risk of colon cancer [7].

At present, the role of the body mass index (BMI) and motilin in the etiologic development of functional constipation is still unclear. The aims of this study were to evaluate the relation between BMI, motilin and the slow transit constipation (STC).

## Patients and Methods

### Patients

A total of 216 subjects had been assessed by a gastroenterologist and had chronic constipation, defined by the Rome II criteria as follows: during at least 12 weeks, which need not be consecutive, in the preceding 12 months presenting two or more of: less than three bowel movements per week, straining at stool more than 25% of the time, passage of lumpy or hard stools more than 25% of the time, sensation of incomplete evacuation for more than 25% of the time, sensation of anorectal obstruction/blockage for more than 25% of the time and manual maneuvers to facilitate more than 25% of the time of defecation. In addition, loose stools are not present, and there were insufficient criteria for the diagnosis of irritable bowel syndrome [8]. Patients with associated medical conditions that might result in constipation (i.e. secondary causes, such as pregnancy, diabetes mellitus, medication, etc.) were excluded. Constipated subjects with STC were diagnosed by a complete medical history, rectal pelvic- floor and anal physical examination and assessment of bowel transit time.

A total of 123 healthy volunteers were recruited as controls. The volunteers were recruited through advertisement. They were interviewed by validated questionnaires [1] and the research nurses about their health status. They did not have any past chronic medical disease including gastrointestinal diseases. No volunteer complained of abdominal pain, abdominal distention, or disturbances in bowel habits. The average defecation frequency was 1 per day. A careful drug history was obtained for each subject to ascertain that none had taken drugs known to influence gastrointestinal motility during the 2 weeks before the study. No volunteer had previously undergone abdominal or pelvic surgery.

Weight, height were measured and BMI were calculated in all subjects who were divided into three groups according to the BMI, group A (BMI < 20), group B (BMI 20-25), and group C (BMI > 25). Informed consents were signed before the study. The Medical Ethics Committee of Sanming First Affiliated Hospital of Fujian Medical University approved the study protocol. The study was performed according to Good Clinical Practice and International Conference of Harmonisation guidelines.

### Assessment of bowel transit time

STC were confirmed by X-ray and colonic motility studies performed in all 178 patients, who had been shown to have long-standing severe constipation. Colonic transit time was assessed through the use of radiopaque markers. In brief, four sets of distinctive radiopaque markers of different shapes and size (circle on day 1, semi-cylinder on day 2, dot on day 3 and cylinder on day 4) were ingested by the volunteers on 4 consecutive days. X-ray of the abdomen was taken on d 5 and 7 to assess the mouth to anal transit and segmental colon transit. Transit in the right, left, and recto-sigmoid colon was calculated by adding all markers seen in these regions on day 5 and 7. Slow total colonic transit was defined as more than 67 hours, the mean transit plus 2SD averaged from published studies [9, 10].

### Blood sample handling and motilin assay

Five ml venous blood was collected from each subject in fasting and one hour after 500 kcal mixed meal on day 1 morning, then was transferred to polythene tubes with 30 µL 10% EDTA and 30 µL aprotinin, allowed to clot in ice and was centrifuged at 4 °C within 30-45 min of collection. Sera was stored at -20 °C until assayed. Cathartic, acid inhibitor and gastric drugs were prohibited, and their own living habits were maintained. Follicle phase was chosen for females, especially the third to tenth day of menstruation cycle.

The stored blood samples were melted under room temperature and were centrifuged with 3500r/min at 4 °C, and then the supernatants were used for test. All tests were performed according to the introduction of the Kit and the published studies [11, 12]. Motilin kit was provided by Beijing East Asia Immunological Technique Research Center. Radioimmunoassay (RIA) and SN-695 B Gamma Counter were used to test the motilin level.

### Statistical analysis

The results were analyzed statistically by analysis of variance, X^2^ and t tests. We used SPSS 11.5 software for statistical analysis. A p value of less than 0.05 was considered statistically significance throughout. Values are given as the mean ± SD.

## Results

A total of 216 patients of chronic constipation were hospitalized in the Department of Gastroenterology Sanming First Affiliated Hospital of Fujian Medical University from May 16, 2005 to August 30, 2009. Thirty eight subjects were excluded due to bowel transit time less than 67 h and rectal pelvic floor and anal physical examination suggested outlet obstruction. Finally, 178 eligible patients were performed in the study, including 77 males, 101 females with average age 58.6 ± 5.3 years old (16 - 80). The mean constipation history in the patients was 7.2 ± 8 years (range from 1 to 40 years). The groups were well balanced in terms of age, gender, weight, and height. No significant differences wereer observed between each groups (p > 0.05) (Table 1).

**Table 1:** Clinical Data of 178 STC Patients and Healthy Controls (Mean ± SD or Percentage)

Group	Slow Transit Constipation			Healthy Controls		
	A (n = 88)	B (n = 41)	C (n = 49)	A (n = 32)	B (n = 51)	C (n = 40)
Males	49.4% (38/77)	23.4% (18/77)	27.2% (21/77)	26.3% (15/57)	40.4% (23/57)	33.3% (19/57)
Females	49.5% (50/101)	22.8% (23/101)	27.7% (28/101)	25.8% (17/66)	42.4% (28/66)	31.8% (21/66)
Age (y)	57.8 ± 4.3	58.2 ± 6.3	59.7 ± 5.1	56.6 ± 3.8	58.3 ± 4.2	59.9 ± 5.3
Height (cm)	172 ± 3.8	169 ± 4.2	171 ± 4.4	170 ± 4.6	172 ± 3.9	171 ± 3.2
Weight (kg)	46.8 ± 3.1	64.5 ± 4.2	79.5 ± 3.6	47.6 ± 3.2	65.7 ± 3.8	80.6 ± 4.1

There was significant difference in the constituent ratio between STC patients and healthy controls (X^2^ = 18.705, p < 0.05). Percentage of group A, B and C in STC patients was 49.4% (88/178), 23.0% (41/178 ) and 27.6%(49/178), respectively, and group A had a higher percentage.

In STC patients, the right segment transit time of group A was significantly higher than that of group B and C (F = 25.969, p < 0.05), but there was no difference between group B and C (p > 0.05). The left segment transit time of group A was significantly higher than that of group B and C (F = 24.604, p < 0.05), but there was no difference between group B and C (p > 0.05). No significant difference was found among the three groups (F = 0.380, p > 0.05) in the sigmoid-rectal segment transit time. The whole gastrointestinal tract transit time of group A was significantly higher than that of group B and C (F = 5.113, p < 0.05), but there was no difference between group B and C (p > 0.05) (Table 2).

**Table 2:** Transit Time in STC Patients (hour, Mean ± SD)

Group	A (n = 88)	B (n = 41)	C (n = 49)
Right segment	32.8 ± 8.2	23.8 ± 8.3	24.4 ± 7.5
Left segment	29.2 ± 7.9	20.8 ± 8.2	21.1 ± 7.3
Sigmoid-rectal segment	18.7 ± 7.7	18.4 ± 7.1	17.4 ± 7.9
Total transit time	85.9 ± 14.9	79.3 ± 14.5	78.9 ± 12.9

Plasma motilin of fasting and one hour postprandial in STC patients of group A was significantly lower than that of group B and C (F = 131.782, p < 0.05, F = 140.401, p < 0.05), but there was no difference between group B and C (p > 0.05). There was no significant difference in the results of plasma motilin of fasting and one hour postprandial among the three groups of healthy controls (F = 1.891, p > 0.05, F = 1.094, p > 0.05). Plasma motilin of fasting and one hour postprandial in STC patients of group A was significantly lower than those healthy controls of group A (t = -23.098, p < 0.05, t= -32.128, p < 0.05). The same results of plasma motilin of fasting and one hour postprandial could be seen in group B and C, respectively (p < 0.05) (Table 3).

**Table 3:** Plasma Motilin in Different Groups (pg/ml, Mean ± SD)

Group	Slow Transit Constipation (N = 178)			Healthy Controls (N = 123)		
	A (n = 88)	B (n = 41)	C (n = 49)	A (n = 32)	B (n = 51)	C (n = 40)
Percentage	49.4%	23.0%	27.6%	26.0%	41.5%	32.5%
Fasting state	219.0 ± 22.2	287.2 ± 31.3	280.7 ± 29.7	328.4 ± 25.1	337.6 ± 20.8	331.8 ± 20.4
Postprandial (1 h)	266.2 ± 25.0	346.6 ± 30.9	334.9 ± 32.1	435.4 ± 26.9	442.7 ± 21.9	442.0 ± 21.2

## Discussion

Functional constipation is a common problem in clinical practice. The constipation prevalence studies conducted to date yield discordant results, with estimates between 2% and 34% [1-2, 13-17]. It is about 6.07%, 4.0% and 3.68% in BeiJing, GuangDong and TianJing of China, respectively [18-20]. According to community population-based epidemiologic study of chronic constipation in Guangdong province, Li et al [19] found that prevalence of constipation is 7.1% (32/451) in the population whose BMI is lower than 18.5, but it is 2.6% (13/502) in the population whose BMI is higher than 25. It is inferred that the slims are more likely to suffer from chronic constipation, and the reason is not clear. Our study found that the percentage of STC was 49.4% in the patients with BMI lower than 20 among all 178 subjects, but it was 23.0% and 27.6% for those with BMI 20 - 25 and higher than 25, respectively, Group A had a higher percentage among the three groups. The reason of higher proportion of STC in the slims is probably that the slims are more likely to be anxious, depressive and more likely to suffer from psychentonia, pressure of work, social dysfunction, emotional instability and insomnia [21, 22]. Furthermore, these factors easily lead to weight loss [6, 19]. High prevalence of emotional distress including anxiety, depression, and social dysfunction in patients with functional constipation has been well reported [23-25]. In addition, a study demonstrated that constipated subjects with STC are associated with more psycho-social distress than those with normal bowel transit [26].

Motilin, one of branched chain polypeptides consisted of 22 amino acids, molecular weight 2700, is mainly secreted by M-cell, which is located in crypt of the proximal end of duodenum and jejunum of mammalian and nerve tissue such as anterior pituitary, pineal gland, cerebral cortex, cerebellum and hypothalamus [27, 28]. Motilin is a brain-gut peptide hormone. It is secreted less when decrease of sleep [29]. It is a potent excitatory agent when applied iontophoretically to neurons in the cerebral cortex and spinal cord [30]. Intraventricular motilin infusion exhibited anxiolytic effect on mice [31] and the levels of motilin elevated in patients treated with antidepressant and neuroleptic drugs [32]. Li et al [33] found that the traditional Chinese medicine QingZhiShu could increase motilin release of migrating myoelectric complex (m.m.c.) III of the small intestine to improve the gastrointestinal movement. It shows that duodenum and jejunum has higher concentration of motilin [34]. Motilin, as an important factor, can promote intestinal segmentation movement by starting intestinal migrating myoelectric complex, contribute to gastrointestinal contents movement by increase of colon motility [35]. The mechanism is to increase intracellular inositol triphos-phate content and calcium concentration [36].

The secretion of motilin is regulated by neuropsychic factors and affected by eating [37-40]. Zhang et al [39] Measured plasma motilin of 21 patients with STC and 33 heathly people in fasting and one hour postprandial, and found that fasting motilin of STC patients was lower than those healthy people, but the difference was not significant (p > 0.05), which is inconsistent with our study. This difference could be considered as different diet structure in different area. Bland and mild diet with multiple soup is popular in Southern China, on contrary, heavy taste of tingling hot and greasiness is more accepted in YunNan, SiChuan and GuiZhou of China, such diet easily leads to constipation in the following day. Moreover, constipation can cause a further decrease in motilin. However, the levels of motilin one hour after meal (morning diet structure of Southern China is similar to the YunNan, SiChuan and GuiZhou ) were lower than normal people, and the difference was significant (p < 0.05), which is consistent with our study. By investigation the changes of motilin of 18 patients suffered from constipated IBS, after drinking and intaking food which was rich in fat, Sjolound et al [40] reported that the motilin was secreted lower than healthy controls. In addition, a recent study reported that patients with a diarrhea predominant bowel habit had higher fasting motilin levels compared to constipated patients [41]. Our result also showed that the levels of motilin in patients of STC were lower than those healthy controls, the difference was significant (p < 0.05). Especially patients with the lowest BMI of group A had the lowest levels of motilin in fasting and one hour postprandial. However, Kuang et al [42] found that there was no significant difference in motilin of 42 patients with outlet obstructive constipation (OOC), which suggested that treatment with motilin or its receptor agonist was unsuitable for patients with outlet obstructive constipation.

In conclusion, we did not survey the epidemiological condition of chronic constipation in the local population and unable to determine the prevalence of chronic constipation in population of low BMI, but we found a higher proportion of low BMI sufferers among the STC patients. The reason may be related to gastrointestinal neurological disorder due to psychiatric or psychological factors that lead to the low release of the plasma motilin.
